# Examining the effects of gaming and guessing on script concordance test scores

**DOI:** 10.1007/s40037-018-0435-8

**Published:** 2018-06-12

**Authors:** Stuart Lubarsky, Valérie Dory, Sarkis Meterissian, Carole Lambert, Robert Gagnon

**Affiliations:** 1 Centre for Medical Education, McGill University, Montreal, Canada; 2 Centre de pédagogie appliquée aux sciences de la santé (CPASS), Université de Montréal, Montreal, Canada

**Keywords:** Assessment, Clinical reasoning, Script concordance

## Abstract

**Introduction:**

In a script concordance test (SCT), examinees are asked to judge the effect of a new piece of clinical information on a proposed hypothesis. Answers are collected using a Likert-type scale (ranging from −2 to +2, with ‘0’ indicating no effect), and compared with those of a reference panel of ‘experts’. It has been argued, however, that SCT may be susceptible to the influences of gaming and guesswork. This study aims to address some of the mounting concern over the response process validity of SCT scores.

**Method:**

Using published datasets from three independent SCTs, we investigated examinee response patterns, and computed the score a hypothetical examinee would obtain on each of the tests if he 1) guessed random answers and 2) deliberately answered ‘0’ on all test items.

**Results:**

A simulated random guessing strategy led to scores 2 SDs below mean scores of actual respondents (Z-scores −3.6 to −2.1). A simulated ‘all-0’ strategy led to scores at least 1 SD *above* those obtained by random guessing (Z-scores −2.2 to −0.7). In one dataset, stepwise exclusion of items with modal panel response ‘0’ to fewer than 10% of the total number of test items yielded hypothetical scores 2 SDs below mean scores of actual respondents.

**Discussion:**

Random guessing was not an advantageous response strategy. An ‘all-0’ response strategy, however, demonstrated evidence of artificial score inflation. Our findings pose a significant threat to the SCT’s validity argument. ‘Testwiseness’ is a potential hazard to all testing formats, and appropriate countermeasures must be established. We propose an approach that might be used to mitigate a potentially real and troubling phenomenon in script concordance testing. The impact of this approach on the content validity of SCTs merits further discussion.

## What this paper adds

Random guessing is not an advantageous response strategy in script concordance testing. Deliberate selection of the ‘0’ response on all test items demonstrates evidence of artificial score inflation, posing a significant threat to the SCT’s validity argument. Limiting the proportion of SCT items with modal panel response ‘0’ can mitigate the effects of an ‘all-0’ response strategy, but could also have adverse effects on content validity.

## Introduction

The Script Concordance Test (SCT) is used to assess a specific aspect of clinical reasoning competence: clinical data interpretation (CDI) under conditions of uncertainty [1]. Its theoretical foundation emerged from the cognitive psychology literature out of a larger debate about the nature of expertise [2]. According to current theories of expertise development, individuals judged to be ‘experts’ in a given field possess neither superior generic problem-solving skills, nor an enhanced capacity for memory retrieval. The real hallmark of an expert is the structured manner in which his or her knowledge is organized in the mind [3]. Expert knowledge in a given domain is interlinked, or ‘chunked’, in such a way as to facilitate its rapid access, as an integrated unit, at the right time and in the proper context.

In medicine, some of these knowledge ‘chunks’ are referred to as ‘illness scripts’ [4]. Illness scripts are bounded networks of medical knowledge that activate automatically in a physician’s mind early on during clinical encounters, guiding subsequent reasoning through a given case [5]. Illness scripts allow physicians to integrate new incoming information with existing knowledge, recognize patterns and irregularities in symptom complexes, identify similarities and differences between disease states, and make predictions about how diseases are likely to unfold [6].

The SCT aims to examine respondents’ illness scripts under a microscopic lens [1, 7]. In an SCT, examinees are asked to render a judgment—generally using a 5-point Likert-type scale—about the direction (positive or negative) and magnitude (strong or weak) of the association between a new piece of clinical information and a given hypothesis (Tab. 1). A ‘0’ response option is available for respondents to indicate their belief that the new bit of clinical data has no effect on the hypothesis provided. To generate scores, examinees’ responses are compared with those of members of a pre-selected expert panel.^1^ Script concordance hinges on an inference that examinees with ‘higher-quality’ illness scripts interpret data and make judgments in uncertain situations that increasingly concord with those of experienced clinicians given the same clinical scenarios [10].

**Table 1:** Example of a Script Concordance Test item featuring 3 questions nested within one case. *Clinical scenario*: You are evaluating a 63-year-old woman with left-sided weakness in the Emergency Department of your hospital

If you were thinking …:	And then you find …:	Your hypothesis becomes …:				
Q1. Brain abscess	Patient had dental work 1 month ago	−2	−1	0	+1	+2
Q2. Stroke	Patient uses a vaginal oestrogen cream once daily	−2	−1	0	+1	+2
Q3. Brain metastasis	Normal non-contrast CT head	−2	−1	0	+1	+2

−2: Ruled out or almost ruled out; −1: Less likely; 0: Neither more nor less likely; +1: More likely; +2: Certain or almost certain

There are, however, few empirical data to support the claim that SCT offers useful insights into the way that medical knowledge is organized into scripts in the minds of examinees. Lubarsky et al. [11] conducted a review of the literature evaluating the construct validity of the script concordance method, following an established approach for analyzing validity data from five categories: content, response process, internal structure, relations to other variables, and consequences [12]. While the authors found moderate to strong validity evidence in several categories, they concluded that the validity of SCT scores is only weakly supported by evidence pertaining to response process, which entails the unearthing of data elucidating the relationship between an assessment’s intended construct and the thought processes and response actions of its examinees [11].

In one computer-based study using the script concordance format, subjects were asked to gauge the effect (i.e. more likely, less likely, or no effect) of new pieces of information on a series of diagnostic hypotheses [13]. Subjects responded significantly faster when they were presented clinical information that was either highly typical or irreconcilable with the given hypothesis than when they were presented information that was merely atypical, suggesting that processing times were influenced by the ‘degree of compatibility’ between new clinical information and relevant activated scripts—in other words, by the strength of association between acquired health-related concepts in the subjects’ minds. SCT investigators have also pointed to the observation that SCT scores consistently tend to increase with increasing level of training to indirectly support their claim that SCT provides valid information about illness script development [7].

Since the publication of the literature review by Lubarsky et al. [11], other investigators have furnished evidence that, in fact, undermines the response process validity of SCT score interpretations [14–17]. For example, as part of a larger examination of the current SCT scoring system, Lineberry and colleagues [16] investigated the effect on SCT scoring of deliberately responding ‘0’ to all items on the test. Based on a re-analysis of data from previously-published work by Bland, Kreiter, and Gordon [18], the investigators demonstrated that a hypothetical examinee who simply endorsed the scale midpoint for every item would outperform most other examinees using the scale as it was intended.

The point Lineberry and his colleagues [16] were trying to make in devising this inauthentic scenario was that the SCT is susceptible to construct-irrelevant response tendencies, however unlikely. As they point out, the possibility that the SCT could be ‘gamed’ in such a manner poses a potentially serious threat to the response process validity of SCT score interpretation, since test-wise examinees are apt to catch on that defaulting to the ‘0’ response would lead to artificial score inflation. However, the results of the Lineberry study, which involved re-analysis of a single set of scores obtained from an SCT using a suboptimal 8‑member panel, need to be interpreted with caution. Prior work on SCT has shown that, in high-stakes settings, at least 15 panel members are required to obtain stable estimates of the reliability of scores, and that reliability becomes severely compromised when panels consist of fewer than 10 members [19].

The present study aims to shed further light on the particular response tendencies exhibited by SCT examinees, and to address some of the mounting concern over the response process validity of SCT scores. Using published datasets from three independent SCTs, each with a score key devised by reference panels comprising 15 members or more, we sought to examine the ‘epidemiology’ of selected response options by SCT examinees, and to verify the legitimacy of the claim that SCT scores are susceptible to the influences of gaming and guesswork. Our specific research questions were:

1. How frequently are individual Likert-type scale responses (−2, −1, 0, +1, or +2) selected by actual SCT examinees?
2. How would a hypothetical examinee selecting random answers to every item on a specific SCT perform compared with an examinee completing the same test as intended?
3. How would a hypothetical examinee deliberately responding ‘0’ to every item on a specific SCT perform compared with an examinee completing the same test as intended?
4. If influences of guessing (as per Research Question #2) or gaming (as per Research Question #3) are detected on a specific SCT, can item manipulation mitigate these influences?

## Methods

### Databases

To conduct our analyses, we used data culled from three independent, previously-published SCT studies [20–22]. In each study, a panel of at least 15 expert members was used to develop the scoring key for the test. Cronbach’s alpha coefficients were considered to be adequate in all three studies. Test characteristics for each of the SCTs analyzed in this study are shown in Tab. 2.

**Table 2:** Test characteristics

	Radiation-oncology [20]	Neurology [21]	General surgery [22]
*Participants*			
*N* clerks	70	8	0
*N* residents (R1–R5)	38	41	202
*N* panellists	42	16	21
*Items*			
*N* cases	30	24	43
*N* questions	70	79	131
*Proportion of items with panel modal response:*			
−2	6%	9%	26%
−1	38%	27%	40%
0	24%	29%	27%
+1	22%	29%	3%
+2	10%	6%	3%
Cronbach’s alpha	0.90	0.79	0.85

### Analyses

To examine the frequency of selection of individual Likert-type scale responses by actual examinees, we calculated the percentage of each answer selected by participants on the three SCTs under investigation.

To examine the influence on scoring of completing an SCT using a guessing strategy, we derived 100 random answer combinations for each of the three SCT datasets using the Excel random function and computed descriptive statistics. For each study, we conducted the analysis once using a traditional 5‑point scale, and a second time using a proposed alternative 3‑point scale [18].

To examine the influence on scoring of completing an SCT using an ‘all 0’ strategy, we calculated the score a hypothetical examinee would obtain on each of the three SCTs if he or she were to consistently answer ‘0’ on all test items.

To examine the feasibility of mitigating the effects of gaming on score inflation, we devised a strategy using data from Lambert et al.’s [20] database, which resulted in the highest mean score for a hypothetical examinee adopting the ‘all 0’ response tactic. Since questions for which the modal panel response is 0 award full credit to examinees who also respond 0, we surmised that reducing their number would be the most efficient way to limit the benefits associated with an ‘all 0’ gaming strategy. We therefore excluded these questions one by one, recalculating the hypothetical examinee’s final score after each item was discarded. For each iteration of the test, we also calculated z‑scores, scores and associated descriptive statistics for actual respondents, and Cronbach’s alpha as an indicator of test reliability.

Given the hypothetical nature of the experiment and the lack of potential to cause harm to human individuals, formal review by our institution’s ethics review committee was waived.

## Results

1. *Frequency of actual responses* (Tab. 3)

**Table 3:** Distribution of responses according to sub-groups of respondents

		−2	−1	0	+1	+2
Radiation-oncology	Clerks	10%	28%	28%	22%	12%
	Residents	9%	27%	22%	28%	14%
	Panellists	11%	27%	29%	20%	13%
Neurology	Clerks	13%	30%	23%	25%	10%
	Residents	10%	30%	23%	25%	12%
	Panellists	10%	28%	31%	23%	8%
General surgery	Residents	19%	38%	23%	14%	6%
	Panellists	25%	34%	23%	11%	7%

In the three SCTs under analysis, the 0‑response represented 21–31% of test answers, with no differences observed according to expertise level (Tab. 3). Response patterns were similar for two of the tests, i.e. the extreme points of the Likert-type scale (−2 and +2) were selected less frequently; most answers were equally distributed between the −1, 0 and +1 points. In the general surgery test, answers were spread mostly across the −2, −1 and 0 points.

2. *Guessing* (Tab. 4)

**Table 4:** Scores of actual respondents, a hypothetical examinee providing random answers (i.e. guessing), and a hypothetical examinee using an ‘all 0’ strategy (i.e. gaming)

	Groups	*N*	Mean	SD	Z score*
*Radiation-oncology*					
Actual respondents	Clerks	70	51.6	8.2	
	Residents	38	71.2	9.5	
	All examinees	108	59.3	9.7	
Guessing	Random 5	100	35.8	4.0	−2.42
	Random 3	100	43.7	2.7	−1.63
Gaming	All 0		52.2		−0.73
*Neurology*					
Actual respondents	Clerks	8	63.6	6.3	
	Residents	41	70.3	8.8	
	All examinees	49	67.9	8.8	
Guessing	Random 5	100	35.9	4.4	−3.6
	Random 3	100	50.9	4.1	−2.0
Gaming	All 0		53.2		−1.7
*General surgery*					
Actual respondents	Residents	202	68.1	7.4	
Guessing	Random 5	100	42.6	3.0	−3.4
	Random 3	100	53.8	3.2	−1.9
Gaming	All 0		51.7		−2.2

*Z scores indicate by how many standard deviations a score deviates from the mean

A simulated guessing strategy based on use of a 5-point Likert-type scale (denoted ‘Random 5’ in Tab. 4) led to mean scores ranging between 2.42 and 3.60 standard deviations below the mean scores of actual examinees. A simulated guessing strategy based on use of a 3-point Likert-type scale (denoted ‘Random 3’ in Tab. 4) led to scores ranging between 1.63 and 2.00 standard deviations below the mean scores of actual examinees.

3. *Gaming* (Tab. 4)

A simulated gaming strategy, whereby a hypothetical examinee was assumed to deliberately answer 0 on every question, resulted in scores ranging between 0.73 and 2.2 standard deviations below the mean scores of actual examinees.

**Fig. 1:**
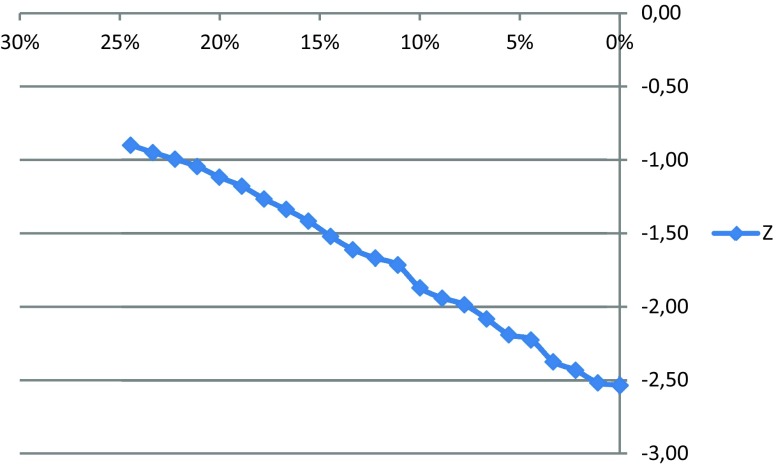
Z-score of a ‘gamer’ on subtests with different proportions of questions with modal panel responses of 0

4. *Mitigating effects of excluding questions with a modal panel response of 0* (Tab. 5; Fig. 1)

**Table 5:** Effect on actual respondent scores (using data from Lambert et al. [22]) of excluding questions with a modal panel response of 0 one-by-one, and recalculating the examinee’s final score after each item was discarded. In shaded rows, a pass-fail threshold of 2 standard deviations below the mean would ensure that gamers fail the test

*N* of questions with a modal panel response of 0	% Questions with a modal panel response of 0	‘All-0’ score for a hypothetical examinee	Mean score of actual examinees	Min	Max	All 0 z score	Cronbach’s alpha
22	24.4%	52.2	62.0	37.3	84.1	−0.90	0.904
21	23.3%	51.0	61.2	37.3	83.0	−0.95	0.902
20	22.2%	49.9	60.5	36.2	81.8	−1.00	0.900
19	21.1%	48.8	59.7	35.8	80.7	−1.04	0.896
18	20.0%	47.7	59.2	35.8	79.6	−1.12	0.893
17	18.9%	46.6	58.5	35.8	78.5	−1.18	0.892
16	17.8%	45.5	57.9	35.8	77.4	−1.27	0.891
15	16.7%	44.4	57.4	35.6	76.3	−1.34	0.891
14	15.6%	43.3	56.8	35.5	75.2	−1.42	0.888
13	14.4%	42.2	56.3	35.4	74.1	−1.52	0.884
12	13.3%	41.0	55.8	34.3	73.6	−1.61	0.882
11	12.2%	39.9	55.1	34.1	72.7	−1.67	0.881
10	11.1%	38.8	54.2	33.7	71.6	−1.72	0.879
9	10.0%	37.7	53.6	33.9	70.8	−1.87	0.876
8	8.9%	36.6	52.9	32.6	70.4	−1.94	0.875
7	7.8%	35.5	52.3	31.5	69.3	−1.99	0.871
6	6.7%	34.4	51.7	31.1	68.2	−2.08	0.868
5	5.6%	33.3	51.4	30.4	68.2	−2.19	0.865
4	4.4%	32.3	50.6	30.0	67.1	−2.22	0.865
3	3.3%	31.0	50.1	30.0	66.8	−2.37	0.860
2	2.2%	29.9	49.3	29.4	65.6	−2.43	0.858
1	1.1%	28.8	48.6	29.2	65.1	−2.52	0.857
0	0.0%	27.7	47.7	28.3	64.0	−2.53	0.859

Excluding questions with a modal panel response of 0 had a linear effect on the z score of a hypothetical examinee using an all 0 strategy, with minimal impact on the internal consistency of the test. Based on calculations from the Lambert et al. [20] dataset, limiting the proportion of questions eliciting a panel modal response of ‘0’ to fewer than 7% ensured that the all-0 strategy resulted in scores below 2 standard deviations from the mean.

## Discussion

In this study, we sought to examine the general response patterns of script concordance test (SCT) examinees, and to explore the potential effects on SCT scores of 1) guessing (i.e. selecting responses in a random fashion) and 2) gaming (i.e. selecting responses in a deliberate manner to manipulate the scoring system). Calculating the scores a hypothetical examinee would obtain if he had engaged in either of these tactics, and comparing these with the scores obtained by actual examinees on several published SCTs, we found several interesting results.

First, we found that in all three SCTs under investigation examinees tended to avoid selecting responses at the extremes of the Likert-type scale (i.e., +2 and −2), a response tendency that has been suspected but never empirically verified. This response pattern was similar for examinees at all levels of expertise (i.e. from clerkship student to practising physician). In general surgery (but not neurology or radiation oncology), both residents and practising surgeons were more likely to select responses on the negative than the positive spectrum of the Likert scale, hinting that response tendencies might vary according to discipline of practice (although test-specific effects, rather than discipline-specific effects, certainly cannot be excluded in this small study). In none of the studies was the 0‑response more frequently selected than +1 or −1 responses; we found no evidence of an all-0 response strategy, or even a predilection toward 0‑responses, in any of the SCTs we examined.

Second, we found that a simulated random guessing strategy using a 5-point Likert-type scale led to scores at least 2 standard deviations (SDs) below mean scores of actual respondents on three separate SCT datasets. If the common cut-off point for normative standard setting, i.e. −2SD from the mean, were to be used as a pass/fail marker, a guessing strategy on a 5-point Likert-type scale would not prove to be advantageous.

A simulated random guessing strategy using a 3-point aggregate Likert-type scale, on the other hand, led to higher scores than those calculated using the 5‑point Likert-type scale. This finding offers a potentially useful contribution to the ongoing discussion over the optimal method for scoring an SCT [18, 23]. Bland and colleagues [18], for example, found few differences in psychometric performance between five different SCT scoring methods (including use of an aggregate 5‑point and aggregate 3‑point Likert-type scale). However, if indeed use of the two scales leads to comparable reliability estimates, our finding would suggest that the 5‑point Likert-type scale should be maintained in light of its greater capacity to *withstand* the influences of random guessing.

Third, we found that a simulated ‘all 0’ strategy, whereby the ‘0’ response was assumed to have been purposely selected as an answer to every item on the test, led to scores at least 1 SD *above* those obtained by random guessing. Although we found no evidence of use of an ‘all 0’ strategy in actual respondents at any level of training, this tactic would appear to be effective: in 2 out of 3 studies it artificially inflated scores beyond the −2 SD point recommended for normative standard-setting, and in one study it resulted in scores similar to those obtained by clerks who completed the exam faithfully. This finding replicates the results of the recent hypothetical experiment conducted by Lineberry and colleagues [16], and indeed poses a significant threat to the SCT’s validity argument.

‘Testwiseness’ is a potential hazard to all testing formats [24, 25]. Appropriate countermeasures for any test, including the script concordance test, must therefore be established. In this study, we found that limiting the proportion of items with a modal panel response of ‘0’ to fewer than 10% of the total number of test items mitigated the effects of the ‘all 0’ strategy in one SCT. Our finding that gaming of the SCT can be thwarted by diligent test construction complements the recent findings of See, Tan and Lim [26], who tackled the problem from a slightly different perspective. Examining the effect of deliberate avoidance of +2 and −2 responses (rather than deliberate selection of the 0 response) on test results, See and colleagues [26] found that increasing the proportion of questions with extreme modal answers to 50% overcame score inflation due to abstention from selecting extreme responses.

The impact on the test’s content validity of restricting items with modal 0‑responses to fewer than 10% of the total test items merits further discussion. Current guidelines for SCT construction already recommend that test developers generate one-and-a-half times the amount of questions they actually intend to use, because a certain proportion of items with poor item-total correlations are expected to be eliminated *post hoc* from the final version of the exam [7, 27]. Removal of an additional proportion of items with modal 0‑responses would entail further reduction in sampling from the test’s content blueprint, potentially compromising the test’s content validity. Deliberate attempts during the test construction phase to limit the number of items expected to yield modal 0‑responses from the panel imposes an added constraint on test development.

Moreover, forfeiting 0‑response items would concede one of SCT’s unique testing properties: assessment of an examinee’s ability to discern relevant clinical data from those that have little to no bearing on a given hypothesis, a skill necessary for coping with the ambiguities of daily practice. Trade-off on content validity resulting from discriminate inclusion and exclusion of test items from a question bank, however, is by no means a challenge unique to script concordance testing, and can be minimized through judicious selection of items and careful consideration of a test’s purpose and target group [28].

### Limitations

To examine the effects of guessing on SCT scores, we devised the rather unrealistic scenario in which a hypothetical examinee guesses the answers to all of the questions on the test in random fashion. Although actual examinees are unlikely to respond to test items in such a completely haphazard manner, the demonstration of benefits in scoring through the use of such an extreme strategy clearly would have undermined the validity of SCT score interpretations. Indeed, the use of a deliberate all-0 response tactic—another extreme, improbable test-taking strategy, originally conceived by Lineberry and colleagues [16]—was found to be advantageous.

This study has several other limitations. Although for the purpose of this study we examined several datasets of scores obtained from SCTs created according to published guidelines, all analyses were conducted retrospectively. Furthermore, we did not study SCTs developed for use in disciplines other than medicine, such as pharmacy [29], veterinary medicine [30], and nursing [31]. Finally, the strategy we developed for offsetting the effects of a gaming strategy was used on a single dataset only, and therefore our conclusion that mode-0 responses should represent fewer than 10% of an SCT’s test items cannot necessarily be extrapolated to any other version of the test. However, we believe that the *approach* we adopted (i.e., stepwise exclusion of 0‑modal items until a −2 SD threshold is attained) has merit, and might be implemented in other cases of SCT to mitigate what we have found to be a real and troubling phenomenon in script concordance testing. The approach could be used to examine the effects of eliminating questions with other modal answers (e.g. questions that elicit +1, +2, −1, and −2 modal responses), as well.

### Future avenues of research

Ultimately, the response process validity of SCT scores requires further exploration. An investigation of examinees’ inclination to avoid extreme responses warrants particular attention: Is this tendency ethnically or culturally mediated, as Lineberry and colleagues [16] have postulated? Is it a discipline-specific predisposition? Or is there simply no meaningful difference in the minds of examinees between the ‘extreme’ (+2 and −2) and ‘less extreme’ (+1 and −1) responses?

Perhaps the tendency to avoid the ends of the scale could be tempered by careful wording of the instructions presented to examinees prior to SCT administration (i.e. simply by encouraging consideration of full use of the scale throughout completion of an SCT). Or perhaps the problem resides in SCT item development, i.e. in the challenge posed by creating items that reliably elicit responses ranging across the spectrum of the Likert-type scale. SCT response tendencies could be investigated in greater detail by asking examinees to submit written justifications for their responses, by asking them to think out loud as they respond to SCT items, or by interviewing them just after completion of an SCT. Phrasing of SCT instructions, as well as differences in wording of the scale anchors, likely have a significant influence on examinee response patterns, but thus far have been only minimally examined and warrant further attention [32].

Finally, in order to further examine the effects of guessing and various gaming strategies (all-0, extreme-avoidance, etc.) on SCT scores, a more authentic simulation of a ‘partial’ rather than a ‘complete’ guessing strategy could be modelled, and prospective studies could be undertaken using tests deliberately developed to contain restricted numbers of items with modal response of 0 and/or higher numbers of items with modal responses at the extremes of the Likert scale. Studies investigating the relationship between SCT scores and other measures of knowledge organization could also shed light on whether the proposed inference between the two is substantiated, providing further evidence for or against the response process validity of script concordance testing.

## Footnotes

1 For each SCT item, a maximum score of 1 is given for the response chosen by most of the experts (i.e., the modal response). Other responses are given partial credit, depending on the fraction of experts choosing them. Responses not selected by experts receive zero. An examinee’s total score for the test is the sum of the credit obtained for each of the questions, divided by the total obtainable credit for the test, and multiplied by 100 to derive a percentage score. Psychometricians support the use of this type of system, referred to as ‘aggregate scoring’ [8, 9].
